# What colour are your eyes? Teaching the genetics of eye colour & colour vision. Edridge Green Lecture RCOphth Annual Congress Glasgow May 2019

**DOI:** 10.1038/s41433-021-01749-x

**Published:** 2021-08-23

**Authors:** David A. Mackey

**Affiliations:** 1 Lions Eye Institute, University of Western Australia, Perth, WA Australia; 2 School of Medicine, University of Tasmania, Hobart, Tas Australia

**Keywords:** Genetics, Neuroscience

## Abstract

Eye colour and colour perception are excellent examples to use when teaching genetics as they encompass not simply the basic Mendelian genetics of dominant, recessive and X-linked disorders, but also many of the new concepts such as non-allelic diseases, polygenic disease, phenocopies, genome-wide association study (GWAS), founder effects, gene-environment interaction, evolutionary drivers for variations, copy number variation, insertions deletions, methylation and gene inactivation. Beyond genetics, colour perception touches on concepts involving optics, physics, physiology and psychology and can capture the imagination of the population, as we saw with social media trend of “#the dress”. Television shows such as Game of Thrones focused attention on the eye colour of characters, as well as their Dire-wolves and Dragons. These themes in popular culture can be leveraged as tools to teach and engage everyone in genetics, which is now a key component in all eye diseases. As the explosion of data from genomics, big data and artificial intelligence transforms medicine, ophthalmologists need to be genetically literate. Genetics is relevant, not just for Inherited Retinal Diseases and congenital abnormalities but also for the leading causes of blindness: age-related macular degeneration, glaucoma, myopia, diabetic retinopathy and cataract. Genetics should be part of the armamentarium of every practicing ophthalmologist. We need to ask every patient about their family history. In the near future, patients will attend eye clinics with genetic results showing they are at high risk of certain eye diseases and ophthalmologists will need to know how to screen, follow and treat these patients.

## 摘要

当教授遗传学时, 眼睛的颜色和颜色的感知是很好的例子, 因为它们不仅包括显性、隐性和X连锁疾病的基本孟德尔遗传学, 而且还包括许多新兴的概念, 如非等位基因病、多基因病、表型复制、全基因组关联研究(GWAS)、创立者效应、基因-环境的相互作用、变异的进化驱动因素、拷贝数变异、插入缺失、甲基化和基因失活。除了遗传学, 颜色感知还涉及到光学、物理、生理学和心理学等概念, 可以抓住人们的想象力, 正如我们在社交媒体上看到的“#the dress”的潮流。《权力的游戏》等电视节目将注意力集中在人物眼睛的颜色, 也包括他们的冰原狼和龙。这些流行文化的主题可以作为工具, 用于教育并鼓励每个人参与遗传学研究, 而遗传学正是现在所有眼病的关键组成部分。随着基因组学数据的爆炸式增长, 大数据和人工智能使得医学发生了转变, 因此眼科医生需要具备一些遗传学知识。遗传学不仅与遗传性视网膜疾病和先天性异常有关, 也是致盲性眼病的主要原因, 包括年龄相关性黄斑变性、青光眼、近视、糖尿病视网膜病变和白内障。遗传学应该成为每一位执业眼科医生知识储备的一部分。我们需要询问每位患者的家族史。在不久的将来, 基因检测结果显示患有某些眼病的患者将前往眼科诊所 (进行诊治), 眼科医生需要知道如何筛查、跟踪以及治疗这些患者。

## Introduction

**Fig. 1:**
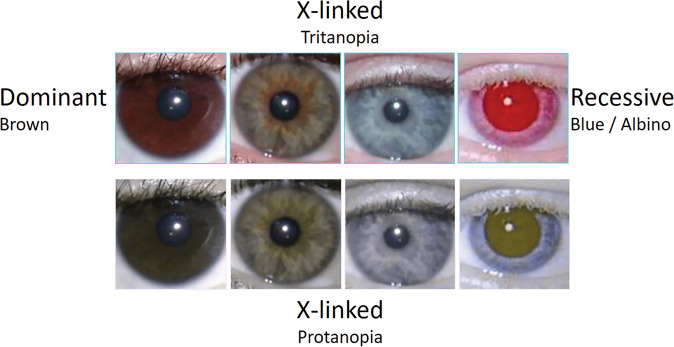
Basic Mendelian Genetics of Eye Colour and Colour Perception. Upper row: Brown, Hazel/Green, Blue and Albino eyes as seen by most of the tritanopic “normal” population. Lower row: the same eyes as would be perceived by a person with X-linked protanopia.

**Fig. 2:**
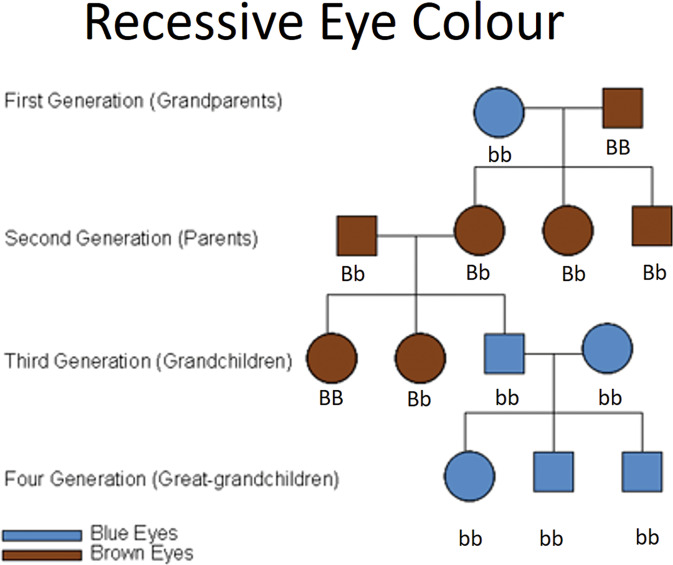
Simple four-generation Mendelian Pedigree of Brown and Blue eyes. Phenotype shown as brown or blue while dominant brown gene = B and recessive blue gene = b Individuals with bb have blue eyes, while individuals with BB or Bb have brown eyes.

Eye colour, or more correctly iris colour, is often used as an example for teaching Mendelian genetics, with brown being dominant and blue being recessive. Colour blindness “Daltonism”, which affects 8% of the male population, is a leading example for teaching X-linked recessive disease (Fig. 1). This simple model works well most of the time, with the main blue eye gene *OCA2*. We can draw pedigrees showing homozygote blue- and homozygote brown-eyed parents having heterozygote brown-eyed children and then grandchildren who may be homozygote or hererozygote blue- or brown-eyed depending on their other parent (Fig. 2).

Blue or brown describes only a portion of eye colour. There are intermediate variations of green and hazel, as well as albino eyes, which lack pigment entirely—all examples for which the simple Mendelian model does not apply. Geneticist Victor McKusick stated, “The early view that blue is a simple recessive has been repeatedly shown to be wrong by observation of brown-eyed offspring of two blue-eyed parents” [1]. This may have inspired his own interest in genetics, as he and his identical twin brother had brown eyes and their parents had blue! We now know that eye colour is actually a complex genetic trait, involving interaction of some major genes and many minor genes. This Mendelian-Complex genetic explanation for eye colour also crosses over into the genetics of many other eye diseases such as age-related macular degeneration and glaucoma. Many people can look at the eye colours in their own families and draw their own pedigrees to see how the Mendelian model applies. Individuals of Asian or African ancestry, most of whom have brown eyes, can still look at other families. Capturing the attention of the public with eye colour and genealogies was done marvellously by the TV series (and books) of Game of Thrones. Viewers tried to predict events based on eye colour, “I see a darkness in you. And in that darkness, eyes staring back at me. Brown eyes, blue eyes, green eyes. Eyes you’ll shut forever. We will meet again.” Melisandre. Game of Thrones season 3.

## More than just blue and brown

### Classification of colour

Whilst the Young–Helmholz trichomatic theory of colour vision (now explained by the presence of three different colour opsin genes) suggests we can define all our perceived colours as mixtures of red, blue and green (or with our colour printers using complementary colours: cyan, magenta or yellow), there is no agreed ‘definition of eye colour’ and there are challenges in consistency of describing colours across different languages and cultures. Researchers of language show that many words for colour have developed differently, the obvious explanation being the environment that people were living in and the objects that they needed to describe. Berlin and Kay identified 11 possible basic colour categories, starting with white/black, red, green/yellow, blue, brown, purple/pink/orange/grey [2]. Physicist Isaac Newton even added two colours to the rainbow—orange and indigo—bringing the total to seven (red, orange, yellow, green, blue, indigo, violet) so that the colours would be divided after the manner of a musical chord [3].

**Fig. 3:**
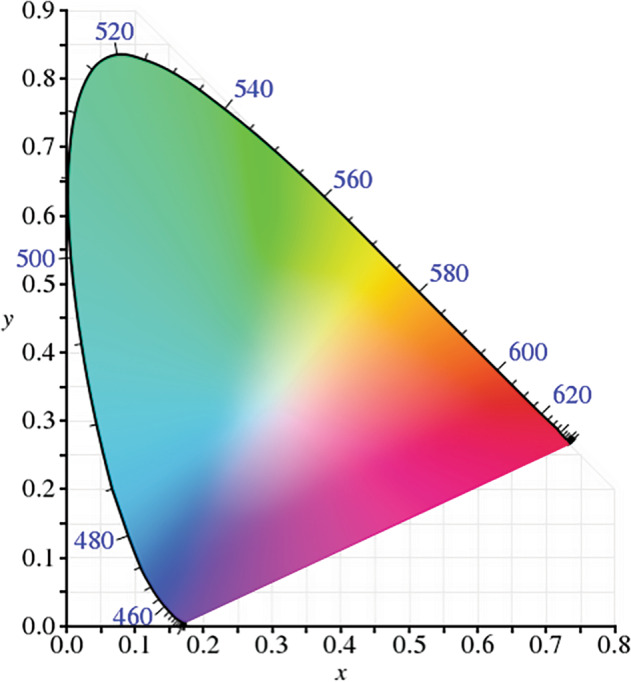
CIE colour diagram. This shows three arms and the complete perceived spectrum of colours.

“Let there be light” Genesis 1:3. Colour is actually a perception rather than a physical property of light so I ask students, “What colour was the big bang?” As no-one was present at the time to perceive it, there was no colour—in fact visible light did not appear until 380,000 years later. Colour can be described in terms of hue (dominant wavelength of the visible spectrum), saturation (the amount of white light mixed in with a hue) and brightness (intensity of light). Any desired hue of light can be produced when various amounts of the three primary colours of light (red, green and blue (RGB)) are combined, either by addition or by subtraction. In 1931, the International Commission on Illumination (CIE) first defined quantitative links between physical pure colours (i.e. wavelengths) and physiological perceived colours in human colour vision [4]. The CIE 1931 RGB colour space (Fig. 3) and CIE 1931 XYZ colour space encompass all perceived colours. This allows one to translate different physical responses to visible radiation in colour inks, illuminated displays and recording devices such as digital cameras, into a universal human colour-vision response.

We now have several major colour systems, including the World Wide Web HTML colours, which are defined using a hexadecimal notation (HEX) for the combination of RGB colour values [5]. The lowest value that can be given to one of the light sources is 0 (in HEX:00) and the highest value is 255 (in HEX:FF). The combination of Red, Green, and Blue values from 0 to 255 gives more than 16 million different colours (256 × 256 × 256). There are also commercial colour-matching schemes, such as the Pantone Matching System [6], that list more than 3000 colours for use in a variety of industries, such as design and manufacturing, in physical and digital formats. They describe more than 50 shades of grey.

### Classification of eye colour

**Fig. 4:**
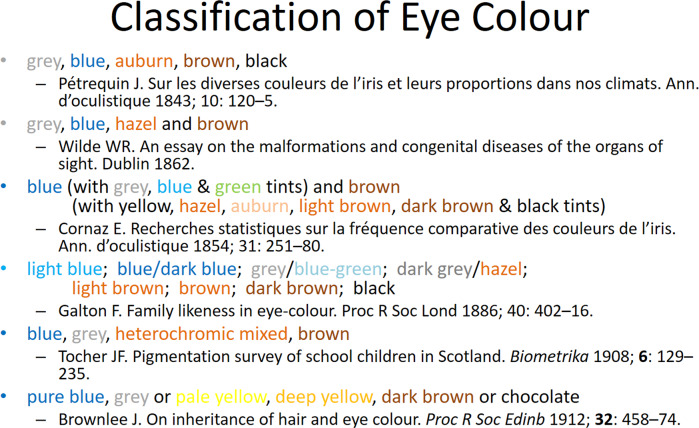
Some early classifications of eye colour with different subgroups. Colour names shaded to the colour mentioned.

**Fig. 5:**
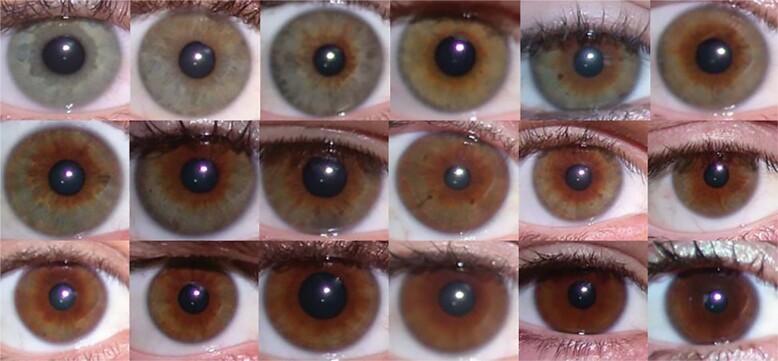
A series of iris photographs. This shows a continuum of green with brown iris colour, ranging from small pupil ring to almost complete brown with peripheral green flecks.

Researchers have been classifying eye colour in either French or English since the nineteenth century [7], initially starting with a small subset of categories (Fig. 4 [8–13]) and, in recent decades, moving more fully into computer-generated classifications [14]. In addition to the overall colour of the iris, in some people there is variation in the distribution of the pigment. In the Twins Eye Study in Tasmania, we documented a continuum of green iris with a small ring of brown pigment around the pupil margin, that was wider in some people, extending through to the periphery with brown eyes with a few peripheral green flecks (Fig. 5). In addition, other structural features of the iris can influence eye colour. These include: iris crypt frequency, furrow contractions and iris naevi and these also have genetic influences [15].

### Where does blue come from?

The main pigment in the eye is the dark brown melanin, whilst the scattering of light from the collagen fibres in the sclera make it appear white and the haemoglobin in the blood vessels appears red. Cyanosis is a bluish colour given by high levels of de-oxyhaemoglobin in the blood, but this is not related to eye colour. So where does blue come from as there is no blue pigment in the eye? The explanation for why some eyes are blue is the same as why the sky is blue, a phenomenon known as the Tyndall effect. Light is scattered by particles in the atmosphere (or by the opaque layers in the iris) with blue scattered more than red. Blue iris is an example of a structural colour rather than a pigment colour. Brown irises have the same layer with more melanin and appear brown while complete absence of melanin (Albinism), the iris appears red from the red of the retina.

## Moving beyond the simple Mendelian model

**Fig. 6:**
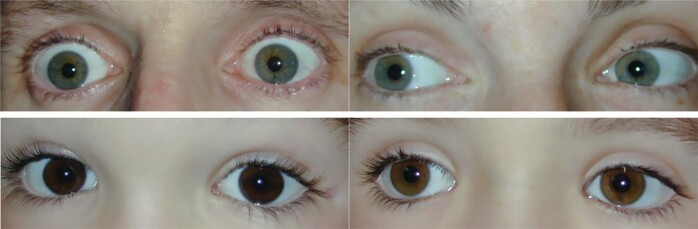
Iris photos of a family. Parents above with blue-green eyes have two children below with dark brown eyes.

**Fig. 7:**
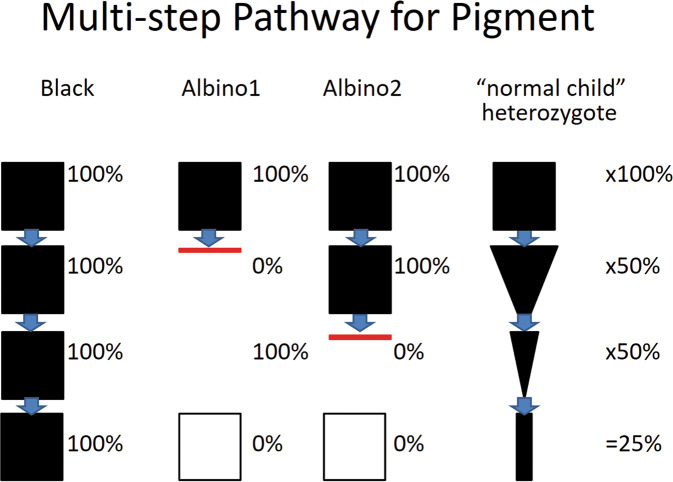
A multi-step pathway for pigment shows that the production of pigment can be blocked at different steps in its production and distribution to the tissues. One parent may be recessive at the first step, while the other parent may be recessive at the second step. Thus, for each step, one of the parents does provide a “normal” copy of the gene needed and thus pigment is produced (although it may be reduced in amount).

Whilst two parents with blue-green eyes may ponder the genetics of their dark-eyed offspring (Fig. 6), the best example of this apparent breach of Mendelian rules was published in 1952 where two parents with oculo-cutaneous albinism had three normally pigmented offspring. These families can be explained if we consider the formation and deposition of pigment as a multi-step pathway and thus it is possible to be recessive at different steps in the pathway. It is possible that one parent had mutations in the *OCA1* (tyrosinase gene) while the other parent had mutations in the *OCA2* gene (Fig. 7). Seven *OCA* genes have been identified (and many more known to influence pigment). If parents are recessive at the same gene, then this is called allelic. However, if there are different genes involved in the development of a single character, these are non-allelic. Another clinical example is Leber Congenital Amaurosis, a monogenic disease for which at least 27 different genes are implicated [16]. If two people with different genetic types of recessive LCA had offspring, then the children would be unaffected carriers (although for two different LCA genes).

### Eye colour in twins, heritability and linkage analysis

**Fig. 8:**
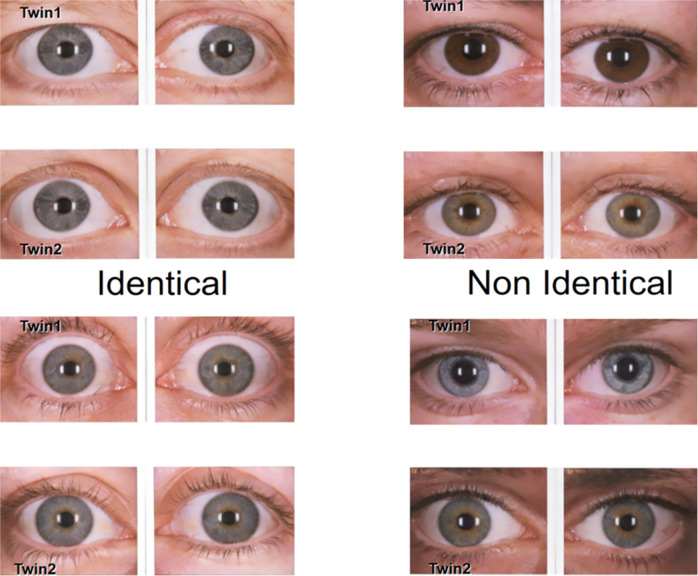
Iris photos from 4 sets of twins; two sets of identical twins on the left and two sets of non-identical twins on the right. Note the increased similarity of eye colour in the identical twins.

Research with twins is a powerful tool in determining heritability. Heritability is the proportion of phenotypic variation in a population that is attributable to genetic variation among individuals. Many eye diseases and measures of ocular biometry, including eye colour (Fig. 8), have high heritability [17]. A study of 920 twin families (389 identical or monozygotic (MZ) and 531 non-identical or dizygotic (DZ)) correlated eye colour in the two groups of twins from Queensland. Statistical modelling suggested 74% of variability of this quantitative trait locus (QTL) was due to a dominant genetic factor (*OCA2*), 18% additive genetic factors (polygenic/other genes) and 8% unique environmental factors [18]. The same study then conducted a genome scan using 382 autosomal and 18 X-chromosomal markers at an average spacing of 9.1 cM on a subset of the twins, Using linkage analysis, a microsatellite marker on chromosome 15q (D15S1002) was identified that was <1 cM telomeric of the *OCA2* gene with a statistical lod of 19.2, which is highly significant. No other gene region met the statistical cut-off of LOD 3. LOD or log of the odds is a statistic used in genetic linkage analysis and the usual cut-off when using several hundred markers in a linkage study (similar to a *P* value of 0.5) was 10 to the power minus 3 or 1/1000.

**Fig. 9:**
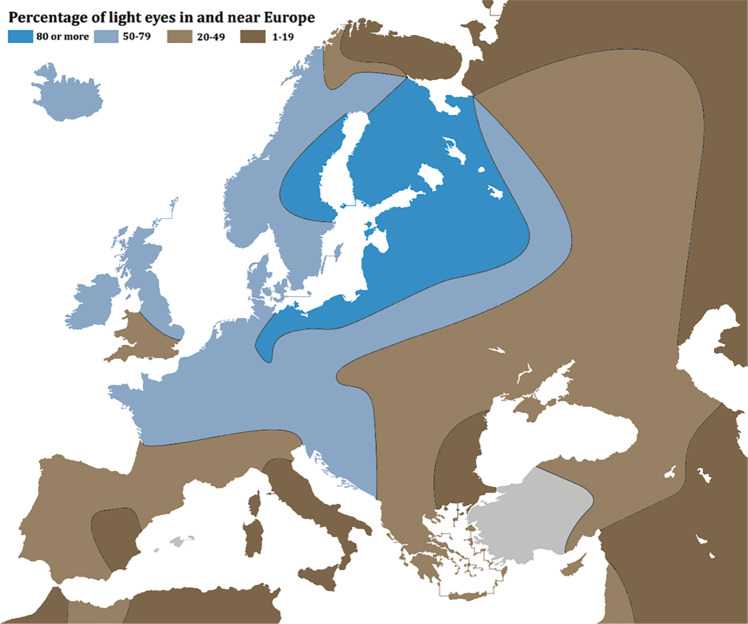
Percentage of light-coloured eyes in Europe. Blue eyes are most common around the Baltic Sea. (creative commons Robert Frost) (https://commons.wikimedia.org/wiki/File:Eye_colors_map_of_Europe.png#filelinks).

A subsequent study looked at this region in more detail using single nucleotide polymorphisms (SNPs) markers in 3839 adolescent twins, their siblings and their parents [19]. The highest association for blue/non-blue eye colour was found with three *OCA2* SNPs: rs7495174 T/C, rs6497268 G/T and rs11855019 T/C in intron 1 of *OCA2*. These three SNPs are in one major haplotype block, with TGT representing 78.4% of alleles. The TGT/TGT diplotype found in 62.2% of samples was the major genotype seen to modify eye colour, with a frequency of 0.905 in blue or green compared with only 0.095 in brown eye colour. A three-SNP haplotype in intron 1 of *OCA2* explains most human eye-colour variation. Finding a segment of chromosome where several identical markers run together in association with a disease or trait suggests that there may be an ancestral founder effect. The highest frequency for blue eyes in the population is around the Baltic Sea and this may be where the original genetic mutation originated [20]. (Fig. 9).

**Fig. 10:**
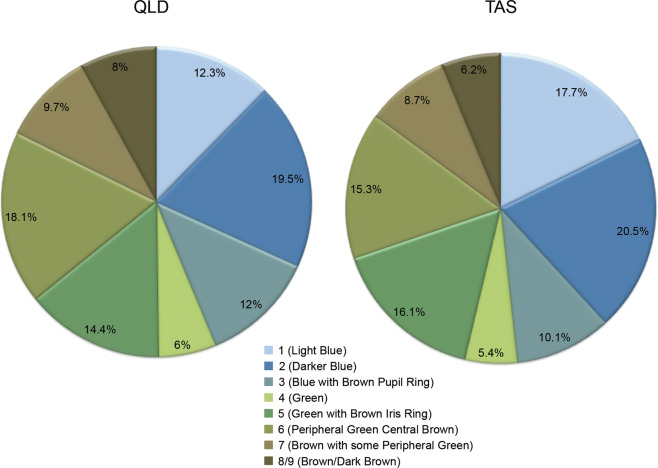
Pie charts showing frequencies of different eye colours from the Twins Eye Study in Queensland and Tasmania. Relative frequencies of different eye colours are shown, with Tasmania having more light blue eyes.

An observation on frequency of eye colour from the Twins Eye Study in Tasmania and Brisbane showed there were more pale blue-eyed people in higher latitude Tasmania compared to subtropical Queensland, suggesting blue-eyed (and fair-skinned) immigrants may have chosen to avoid the sun [21] (Fig. 10).

### Genome-wide association studies (GWAS) for eye colour

With the development of DNA SNP markers, we were able to move from genome-wide scans of hundreds of DNA markers to hundreds of thousands of DNA markers and thus identify many more genes associated with disease. Because we are looking at so many SNPs at the same time, the statistical significance values in GWAS studies were thus higher than previous linkage studies with 10 to the power minus 8 being the usual cut off. In addition, larger studies became available, and researchers could combine their studies in larger meta-analyses. GWAS on 5951 Europeans from the Rotterdam Study identified three new regions, 1q42.3, 17q25.3, and 21q22.13, with the latter two loci replicated in 2261 individuals from the UK and in 1282 from Australia: [14] the *LYST* gene at 1q42.3 and the *DSCR9* gene at 21q22.13. In addition, nine previously identified genes (*HERC2/OCA2, SLC2A4, TYR, TYRP1, SLC45A2, IRF4, NPLOC4, KITLG, MC1R*) were linked.

**Fig. 11:**
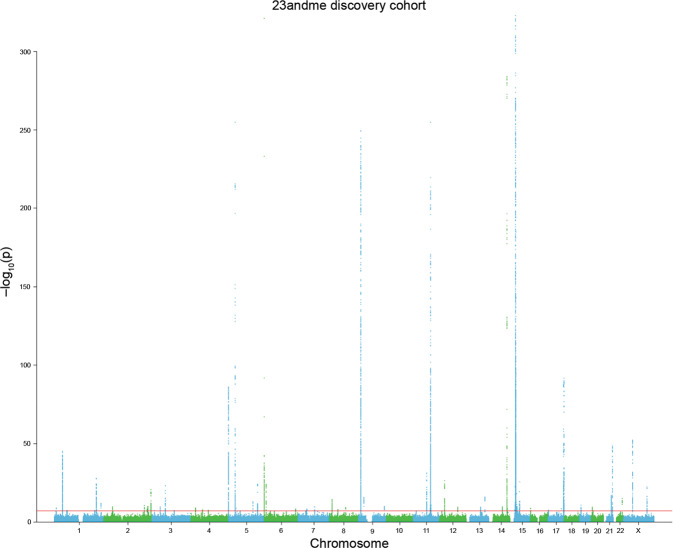
A Manhattan plot for GWAS of eye colour. Each SNP in the study is shown in relation to its chromosomal location on the *X*-axis and the *p* value on the *Y*-axis. The *p* values > 8 are statistically significant, with some highly significant genes resembling Manhattan skyscrapers. Image provided by Mark Simcoe from data in reference [22] Simcoe et al.

An even bigger GWAS study in almost 195,000 individuals from 10 populations identified a further 50 previously unidentified genetic loci for eye colour [22]. (Fig. 11) These genes included those involved in melanin pigmentation, but there were also associations with genes involved in iris morphology and structure. Further analyses in 1636 Asian participants from two populations suggest that iris pigmentation variation in Asians is genetically similar to Europeans, but with smaller effect sizes. The currently identified genes explain just over 50% of eye colour variation using common SNPs. The genetic complexity of human eye colour considerably exceeds previous knowledge and expectations, highlighting eye colour as a genetically highly complex human trait. In terms of genetic epidemiology, we are more certain that *OCA* is associated with blue eyes than any other gene and trait (−log10(p) = 43,740) Hysi personal communication.

### Utility of understanding the genetic architecture of iris colour

The application of these genetic data is particularly useful in forensic and anthropological work, where the eye colour of individuals can be predicted based on DNA evidence. Researchers have inferred that early Neolithic Britons had brown eyes, and that the famous Cheddar Man probably had blue/green eyes, with dark brown (possibly black) hair and dark or dark to black skin [23]. Eye colour is also a risk factor for eye disease; however, researchers can make interesting associations that are more linked to behaviours than genetics such as the gene for blue eyes is common in people who are bad at using chopsticks [24].

## Environmental factors influencing eye colour

### Eye colour change with age (but this could still be genetic)

The twin studies showed that some environmental factors are associated with eye colour [18]. There is surprisingly little published data on change in eye colour with age. Parents are aware that babies’ eyes can darken in the first years of life. The Newborn Eye Screening Test study in California [25] enrolled 202 newborns, of whom 148 were followed up (73% of parents responded at the 2-year follow-up). Brown was the most common iris colour (52.0%) and was less likely to change over time compared to non-brown iris colours (brown to brown, 94%, 73/77). There was a higher frequency of change from blue to non-blue iris colours (blue to brown 27%, 11/40, blue to hazel 7.5%, 3/40 and blue to green 5%, 2/40; *p* < 0.001). Regarding race, at birth, the prevalence of blue irides was significantly higher among White/Caucasian, Native Hawaiian or Pacific Islander, indicating a significant difference in distribution of iris colour between races. Iris colour did not change over the 2-year follow-up period in most cases (66.9%), and only the iris colour of 3.4% (5/148) of participants became lighter from brown to hazel/green, from partial heterochromia to blue and from complete heterochromia to blue.

The Louisville Twin Study [26] (*n* = 1513 individuals) found there was a high degree of concordance in eye colour among identical (MZ) twin pairs, (*r* = 0.98 [*P* < 0.001]), while the concordance was less pronounced in fraternal (DZ) twin pairs (*r* = 0.49) and decreased with age (*r* = 0.07). Eye colour stabilises by 6 years of age in most children but continued to change throughout adolescence and until adulthood in a subpopulation of 10–20% of twins. This suggests that such changes in eye colour, or the propensity to such changes, may be genetically determined. As people age, changes in colour can occur, possibly best exemplified by *Sharbat Gula “The Afghan Girl”* photographed by Steve McCurry in 1984 and again in 2002 for National Geographic.

### Eye colour change with disease

Several diseases are associated with loss of pigment, which is most obvious when only one eye is affected. Changes in iris colour may reveal signs of pathology such as neurofibromatosis, Down syndrome, herpes simplex, pigment dispersion, albinism or primary melanocytic tumours of the iris [7]. Horner syndrome occurs with sympathetic nerve damage to the eye lacking pigment, having a smaller pupil and a drooping lid. Eye Injuries can affect the pupil dilation and the iris pigmentation. A striking example was singer David Bowie. Inflammation from infections such as Fuchs Heterochromic Cyclitis or Herpes Simplex Iritis can result in loss of pigmentation. Naevi, melanomas, and Lisch nodules can increase pigment in lighter eyes while Brushfield spots in some normal people and people with Down syndrome can add white patches to the iris. The interest in loss of eye colour was another aspect of the HBO series Game of Thrones: Eddison Tollett: “Stay back, he’s got blue eyes!” Tormund: “I’ve always had blue eyes!” From Game of Thrones—Season 8 Episode 1: ‘Winterfell’

### Colour perception and Daltonism

Young–Helmholz Theory of Trichromatic Colour Vision is explained by three colour opsins present in the cone cells: Red/Long on the distal end (telomere) of the X-chromosome, Green/Medium also on X and the Blue Short on chromosome 7.

Before the rise of oxygen-producing bacteria, 3750–2500 million years ago, the atmosphere  was nitrogen and carbon dioxide, and so the sky possibly apeared orange. 543–490 million years ago the early Cambrian Explosion saw mass diversification of complex organisms over a relatively short period of time. There was an “evolutionary arms race driven by colour vision”. Modern mammals, evolved after the dinosaur extinction, were mainly nocturnal and did not need colour vision [27]. Humans, apes and Old World monkeys are trichromatic [28]. Why do primates have colour vision? It is thought to be advantageous for the long-range detection of either ripe fruits or young leaves, which frequently flush red in the tropics, and are a critical food resource when fruit is scarce [29].

Dalton, the famous chemist, described his own colour blindness in 1794 [30]. Like his brother, he confused scarlet with green and pink with blue. He thought that his vitreous humour was tinted blue, selectively absorbing longer wavelengths. He instructed that his eyes should be examined after his death, but the examination revealed that the humours were perfectly clear. DNA was later extracted from his preserved eye tissue, showing that Dalton was a deuteranope, lacking the middle wave photopigment of the retina. This diagnosis is shown to be compatible with the historical record of his phenotype, although it contradicts Thomas Young’s belief that Dalton was a protanope [31]. In 1876, Horner gave the first scientific analysis of the hereditary transmission of Daltonism. ‘Horner’s law’ says that colour-blind fathers have colour-normal daughters; and these colour-normal daughters are the mothers of colour-blind sons. In one pedigree, colour blindness is transmitted from the grandfather to the grandson. In a second pedigree, transmission was possible via female carriers through more than one generation. The similarity with the inheritance of haemophilia was mentioned by Horner [32].

### X-linked inheritance was discovered from eye colour in fruit flies

**Fig. 12:**
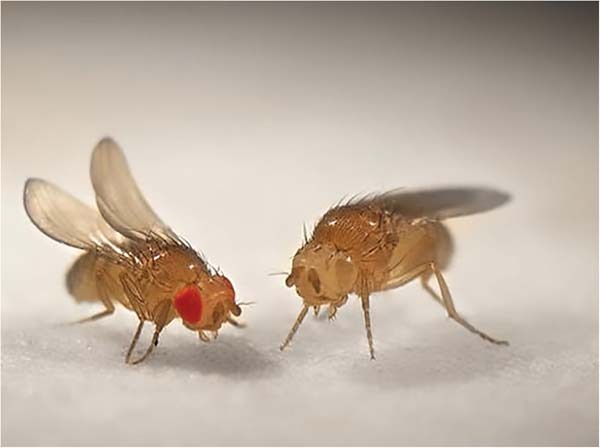
Drosphila melanogaster showing red-eyed (normal) and white-eyed (mutant) variants as identified by Morgan. (creative commons Joe Jimbo) (https://theconversation.com/animals-in-research-drosophila-the-fruit-fly-13571).

Although Mendel published his work in 1866, it wasn’t rediscovered until 1900, and then it only described dominant and recessive inheritance. It was American geneticist Morgan in 1910, working on fruit flies (Drosphila melanogaster), who first described sex linkage [33]. The phenotype of interest was the colour of the eyes! Instead of the normal brilliant red eyes of wild type Drosophila, he identified a fly with white eyes [34] (Fig. 12 [35]).

He performed a test cross between the white-eyed male fly and several purebred, red-eyed females to see whether white eyes occurred in the next generation. The resulting F1 generation all had red eyes; however, Morgan suspected that the white-eye trait was still present but unexpressed in this hybrid generation, like a recessive trait would be. To test this, Morgan then crossed males and females from the F1 generation and observed a 3:1 ratio of red eyes to white eyes in the F2 generation. This result is very similar to those reported for breeding experiments for recessive traits, as first shown by Mendel. However, all the white-eyed F2 flies were male, just like their grandfather—there were no white-eyed females at all! Morgan would be the first person to link definitively the inheritance of a specific trait with the X-chromosome. The mutations for white eye in drosophila is due to ABC transporters that determine eye colour [36].

### X-Linked recessive inheritance

**Fig. 13:**
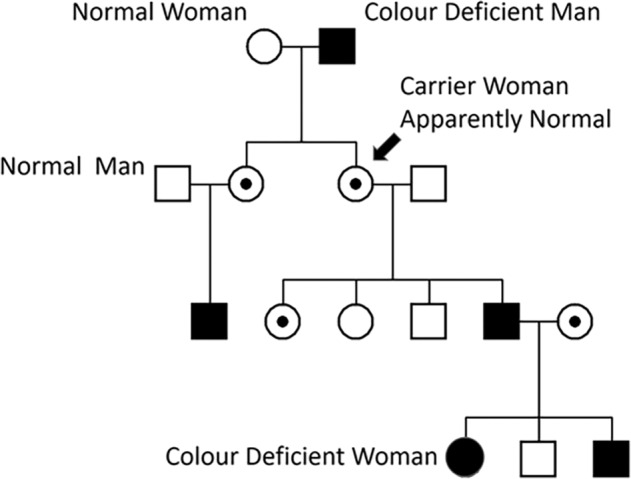
X-linked pedigree showing transmission of colour blindness. Affected males are dark-coloured square, while obligate carrier females as shown as centre-filled circles and affected females as dark full circles.

Men have only one X-chromosome and thus if the X-chromosome carries a mutation, the man will be affected. His daughter will be an obligate carrier, inheriting the mutation from the father but having normal colour vision if she inherits a normal X-chromosome from the mother. The father’s sons only inherit the X-chromosome from their mother, so cannot inherit an X-linked disease from their father. (Male-to-male transmission of a trait is usually regarded as a way of excluding X-linkage). The carrier daughters will have half their sons affected and half their daughters affected. Given that colour blindness (combining the main subgroups of protanopia, protanomaly, deuteranopia and deuteranomaly) affects around 8% of men (and 8% of women are carriers), then occasionally an affected male and a carrier female will produce an affected daughter (8% × 8% = 0.64% of women) (Fig. 13).

### X-inactivation

**Fig. 14:**
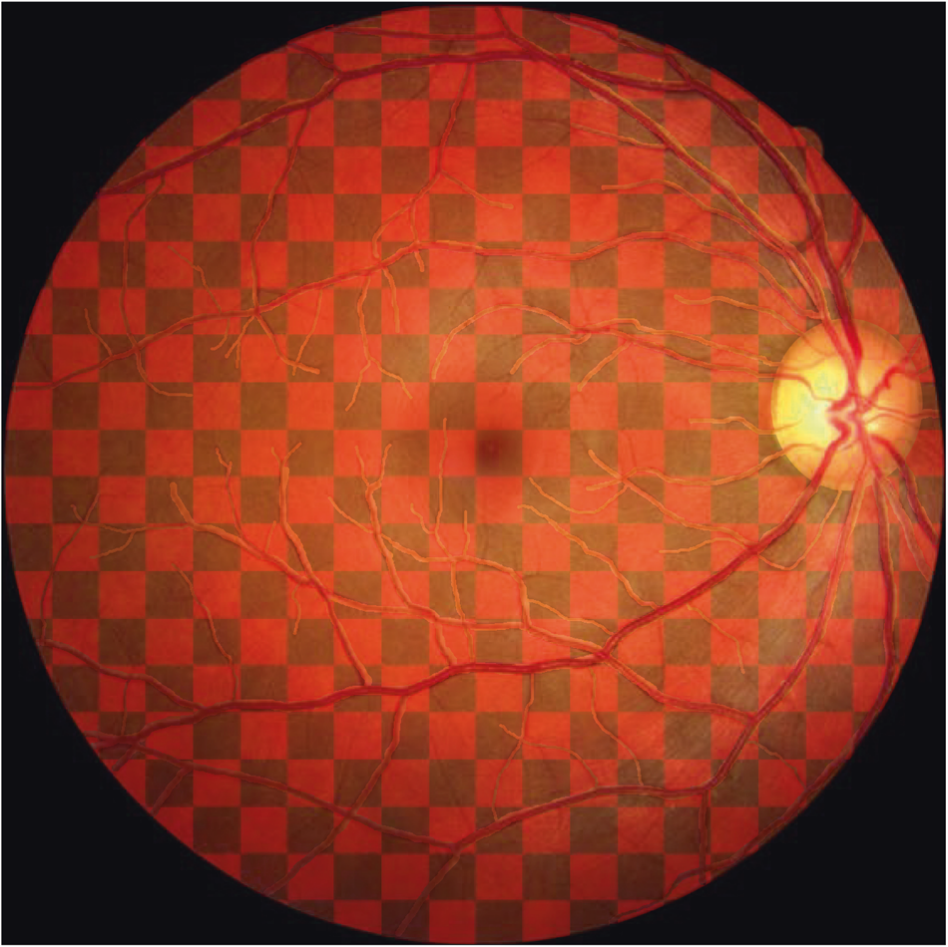
Retinal image with checkerboard patchwork simulating carrier female protanope. These would be less geometrically shaped in real life. Areas in black would perceive a red light as black (protanopic from affected father or carrier mother) while areas in red would perceive red light as red (normal tritanope from normal parent).

In 1961, Lyon described the hypothesis that one of the two X chromosomes of the female is inactivated at an early stage of embryogenesis or X-inactivation. Thus, in X-linked female carriers of colour blindness (and of many other X-linked retinal diseases such as retinitis pigmentosa or choroideraemia), the retina is normally a mosaic of normal and colour anomalous X-chromosome expression. If one passes a coloured laser across the retina, a carrier female may notice a change in colour as the light moves across the mosaic of normal and colour-blind regions (Fig. 14). Rarely, X-inactivation can occur very early and the entire retina expresses the chromosome of only one parent. There are dozens of cases of identical MZ female twins, one of whom is colour blind and the other has normal colour vision [37]. This is an example of epigenetics (methylation) where other modifiers influence the expression of a phenotype and this occurs in many other autosomal diseases.

### Telomeric location of the long (red) and medium (green) opsin allows duplication and rearrangement

**Fig. 15:**
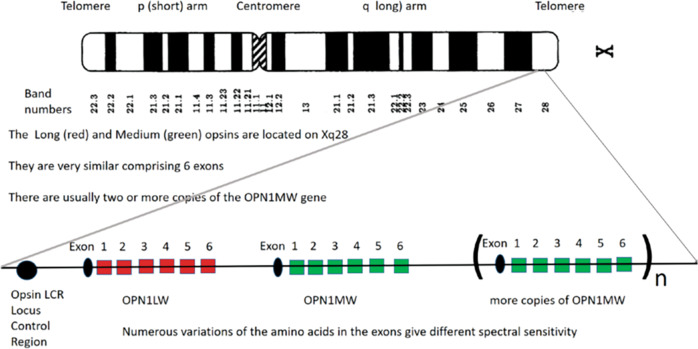
Diagram with location of the colour opsins on the X chromosome. Above a banded X chromosome showing the location of the red (OPN1LW) and green (OPN1MW) opsins in tandem on the telomere of the X-10.1038/s41433-021-01749-x chromosome. Below a OPN1LW gene comprised of 6 exons followed by two OPN1MW genes comprised of 6 exons.

The L (*OPN1LW*) and M (*OPN1MW)* opsins are located at the end of the long arm of the X-chromosome (Xq28). These very similar genes evolved from duplications of a single gene and subsequent mutations and cross-over events creating hybrid genes to a selection of genes, each with different peak spectral sensitivity. Alterations in the cone opsin genes underlie inherited colour-vision deficiencies. Among individuals with normal colour vision, there is variability in the number or  homology of photo-pigment genes and their arrangement in a tandem array allows for a high mutation rate (Fig. 15). Many of the genotypes associated with colour-vision deficiencies are the result of a series of mutational steps away from ancestral gene arrays [38]. Polymorphisms in the L and M opsins give slight differences in spectral sensitivity. Blue cone monochromacy occurs when all the X chromosoma opsins are missing. Other genes also affect the expression of the opsins and the array of the L, M and S photoreceptors [39].

### Variation on rates of colour blindness in men

Some populations, such as the Australian Aborigines, have low rates of red-green colour blindness on Ishihara testing (1.6%), in contrast to 37/503 (7.4%) Europeans in Western Australian regional areas, with similar low rates in Papuans (2%) and Fijians (0.8%) [40]. Why are so many men colour “blind” [41] in most populations? Although well known by the military, there is surprisingly little published information on the ability of colour-blind men to see through camouflage [41]. We conducted a study in Australia showing that pairing people with normal colour vision with those who were colour-blind gave the best results in a search and rescue training exercise [42]. Thus, it is possible that groups of men hunting that included some who were colour blind and some who had normal colour vision may have had a survival advantage for hunter gatherers. A red rag to a bull would appear black. There are some clever adaptations to using colour blindness in pets, such as cats wearing brightly coloured collars that can be used to protect native birds [43].

### #The Dress

**Fig. 16:**
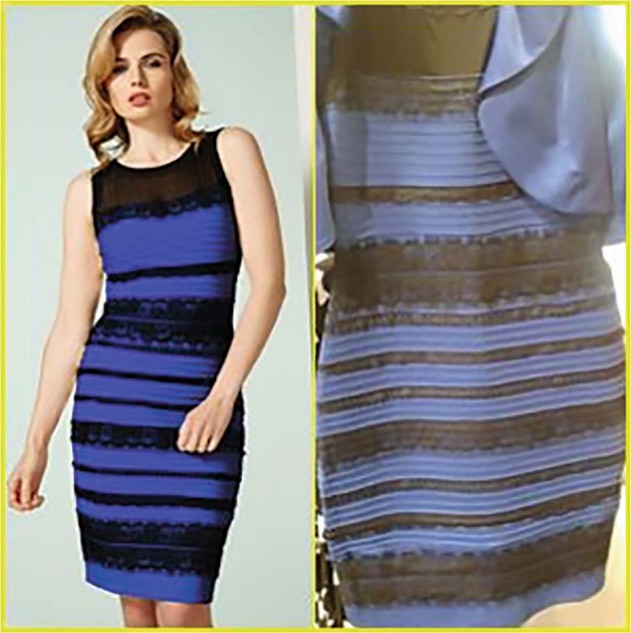
#The Dress of viral internet fame. Actual colour black and blue on the left and the ambiguous image perceived as white and gold or black and blue on the right.

On 27 Feb 2015, a picture of a dress went viral on social media after Celia Bleasdale sent her daughter Grace of Colonsay, Scotland, a photo of a black and blue dress she was going to buy Fig. 16. “That’s lovely mum. The gold and white one.” Celia’s partner also saw the photo as white and gold—even though he had seen the original dress. The daughter shared the picture on Facebook and on February 26, 2015, a friend Caitlin McNeil reposted it on Tumblr, which was noted by Buzzfeed. Buzzfeed got 39 million hits; Tumblr was getting 840,000 views a minute; Twitter had 11,000 tweets per minute! A significant proportion of the population saw it as golden and white, while another significant proportion saw it as blue and black. #The Dress that alerted the world to colour, colour perception and human variation launched over 25 journal articles ranging from explanations of the concept of colour constancy through to genes and environmental experiences [44–66]. Although colour constancy is certainly involved, there is still no theory available to explain the dichotomous character of this optical illusion. Assumptions about the illumination of the dress—i.e. whether the stimulus was illuminated by natural or artificial light or whether it was in a shadow—strongly affects the subjective interpretation of observers [62]. The perception of colours is guided by the scene interpretation and the inferred positions of light sources. People who perceive the dress as blue-and-black are two times more likely to consider the light source coming from the front than those who see the white-and-gold dress. Participants’ environmental lighting conditions are an important cue for perceiving the dress colours [46].

Researchers at 23andMe conducted an instant genetics experiment by asking customers, who have consented to research, about what colours they saw looking at that dress and about 25,000 people responded [67]. The colours seen in those stripes are influenced by the age of the viewer and where the viewer grew up. Those individuals 20 years of age were split evenly between white & gold vs. blue & black, with the proportion seeing white & gold increased up until the age of 60 years (75%). Those who grew up in more urban areas saw white and gold at higher proportions than those who grew up in more rural areas. 23andme found no clear genetic association with seeing either a blue & black dress vs. seeing a white & gold one. A small effect size was noted for a genetic variant in the gene *ANO6*, a member of the anoctamins gene family that includes the gene *ANO2* (expressed in the retina and several brain regions), which is “involved in light perception”. Identical twins also reported seeing different colours. The British TwinsUK registry found MZ twins were more concordant in their responses than DZ twins (0.46 vs. 0.36). Twin modelling revealed that genetic factors accounted for 34% of variation in the reported colour of the dress when adjusted for age, whereas environmental factors contributed 66% [57]. Eye conditions did influence the colour perception: individuals with cataracts were about 50% more likely to see black & blue instead of white & gold; individuals who were colour-blind were more likely to see white & gold but age-related macular degeneration appeared to have no influence. A person’s initial impression of the dress colour was likely to stick. The Dress image illustrates the complex interaction between both our genetics and environment.

## Conclusion

### Colour is in the eye of the beholder

What colour are your partner’s eyes? (Please proceed with caution as not all people give the right answer!) Eye colour and colour perception are very good examples to teach the general population about basic genetic (Dominant, Recessive and X-linked) as well as more complex genetics including: polygenic inheritance, gene duplications and deletions. epigenetics, linkage, genome-wide association studies, founder effects and evolution. Genetics is involved in all the leading eye diseases, not just Inherited Retinal diseases and congenital abnormalities but also the leading causes of blindness: age-related macular degeneration, glaucoma, myopia, diabetic retinopathy and cataract. A good working knowledge of genetics should be part of the clinical skills of every practicing ophthalmologist. We need to ask every patient with any eye disease if they have a family history. “Does anyone else in the family have….?” In the near future asymptomatic patients will attend eye clinics with genetic results that show they are at high risk of certain eye diseases and ophthalmologist will need to know how to screen and follow these patients. Genomics, big data and artificial intelligence will be driving this research in the coming years. We need to equip everyone to understand and utilise this revolution of information by understanding genetics better. From this we will deliver even better outcomes to eradicate blindness and vision impairment.
